# Cognition of Nurses in Neurology Department on Rehabilitation Nursing

**DOI:** 10.1515/tnsci-2019-0005

**Published:** 2019-04-23

**Authors:** Qie Liu, Xin Xu

**Affiliations:** 1 Department of Neurology, Daqing Oilfield General Hospital, Daqing 163001, China

**Keywords:** Cognition, behavioral levels, Neurology, Rehabilitation Nursing

## Abstract

Stroke has become a major disease seriously threating human health due to its high morbidity, mortality and disability. Rehabilitation nursing care for stroke patients has always been a key part of clinical care. The neurological nursing managers should pay high attention to the issue about how to more effectively improve the level of nurses’ rehabilitation nursing on stroke patients. Therefore, this paper investigates the current cognition of neurological nurses about stroke knowledge, attitude and behaviour, and then analyses the factors affecting the knowledge, attitude and behaviour of stroke in the nurses, in order to provide better nursing services for stroke patients, and improve their nursing quality. The findings show that the different cognitions of nurses about their role have different effects on the knowledge, attitudes and behavioural levels of the neurological nurses; the nurses with more types of roles have better knowledge and behavioural levels of stroke.

## Introduction

Stroke has become a major disease seriously threating human health due to its high morbidity, mortality and disability. Stroke patients with hemiplegia are often associated with muscle weakness, muscle spasm and poor intermuscular coordination. Among them, the poor coordination between muscles is mainly shown in the abnormal joint activation pattern. If neurosurgical nurses can understand the biomechanical mechanism of upper limb dysfunction in stroke patients and combine with clinical manifestations, they can provide more targeted Rehabilitation nursing care for stroke patients.

For stroke patients, rehabilitation nursing is a basic content to promote the rehabilitation of the disease. In the implementation of the overall rehabilitation program, the nursing staffs needs to be in the patient in order to obtain comprehensive rehabilitation of the stroke patients in occupation, society, spirit and body. While implementing basic nursing, we will closely cooperate with relevant rehabilitation professionals and rehabilitation doctors to perform functional rehabilitation nursing. However, in the rehabilitation and nursing work of actual stroke patients, there are still many misunderstandings and problems in nursing. In order to find out the causes of these nursing problems, corresponding solutions are formulated to promote the early recovery of patients.

On the other hand, with people’s health awareness gradually improved, patients have put forward higher requirements for the nursing quality. Rehabilitation care for stroke patients is taken in both physical and psychological aspects. So, nurses should be proficient in various rehabilitation knowledges if they want to provide better rehabilitation care for patients ^[1, 2]^. Stroke rehabilitation care is a basic content of stroke rehabilitation. It refers to the functional nursing in addition to the basic nursing for the patients under the guidance of hospital doctors and rehabilitation professionals to achieve the comprehensive rehabilitation of the physical, mental, occupational and social aspects of stroke patients in the process of implementing the rehabilitation program ^[3, 4]^. Due to the existence of many nursing problems and certain misunderstandings during this process, the study conducted a questionnaire survey on neurological nurses, and then understood the nurses’ cognition of relevant theoretical knowledge and practical skill, in order to promote the effective development of stroke rehabilitation and nursing work.

## Research objects and methods

### Research object

A total of 260 nurses in neurology department at four top three hospitals were selected using the cluster sampling method. It included 174 females and 86 males, with an average age of about 30 years. Their academic qualifications were 122 undergraduate degree, 92 college degree, and 46 secondary school educations. In terms of professional titles, it included 42 deputy director nurses, 86 supervisor nurses, 50 senior nurses and 82 nurses; in terms of job titles, there were 206 senior nurses and 54 head nurses. They have over 2-year nursing service in neurology department, with the average nursing age of 19.5 years. Also, the 260 respondents have not received any training courses on stroke rehabilitation nursing knowledge.

### Research methods

Rehabilitation nursing staff (2), rehabilitation therapist (3) and rehabilitation medicine professor (2), based on the combination of stroke rehabilitation nursing knowledge, with reference to a large number of stroke rehabilitation nursing literature, design stroke Rehabilitation Nursing Cognitive Questionnaire. At the same time, from April to May 2010, the survey questionnaire was used to pre-investigate 30 nurses in our department. According to the survey results, the contents of the questionnaire were continuously added and deleted. The survey validity and reliability were 0.87, 0.76. The questionnaire included 20 items related to stroke rehabilitation nursing: the concept of stroke rehabilitation nursing, the connection and difference between general clinical nursing and stroke rehabilitation nursing, and the choice of period of stroke rehabilitation nursing. Each content has 3 answer options: don’t know, don’t know, know.

The questionnaire was distributed and collected by two nurses participating in the survey. At the same time, the head nurse was entrusted to assist in the work. The questionnaire was received immediately and 50 questionnaires were effectively collected. The effective rate of questionnaire recovery was 100.0%.

The questionnaire survey method was adopted in this study. Based on the literature review, by consulting a number of nursing experts related to stroke, the general questionnaires were prepared and the stroke knowledge, attitudes, behaviour questionnaires were made. Thus, after repeated modification, the pre-questionnaires for neurology nurses were finally formed.

### The project analysis of the questionnaire

This questionnaire consists of 19 items at four dimensions: general knowledge, prevention knowledge, risk factor management knowledge, and early rehabilitation exercise knowledge of stroke. All of them are multiple choice questions. There are 4 single choice questions, and 15 multiple choice questions. 4 points are given for each single choice question. For multiple choice questions, 1 point is given for each correct choice; 1 point is deducted for one wrong choice, then, the minimum is 0 and the maximum is 5 points. The higher the score, the better the knowledge of stroke knowledge is (Table 1).

**Table 1:** Project analysis results of each item of neurological nurse stroke knowledge questionnaire (n=240)

Entry	High grouping	Low grouping	t	p
1	2.63±0.60	1.84±0.96	3.05	0.005**
2	1.58±0.69	1.05±0.70	2.32	0.026*
3	1.89±0.57	1.05±1.03	3.13	0.004**
4	2.95±0.23	2.21±1.03	3.13	0.004**
5	4.52±0.61	2.84±1.07	5.97	<0.001**
6	4.21±0.54	2.26±0.93	7.89	<0.001**
7	4.74±0.45	3.32±1.16	4.99	<0.001**
8	3.53±0.84	2.16±0.83	5.04	<0.001*
9	3.79±0.92	1.47±1.98	4.62	<0.001**
10	1.26±1.91	1.05±1.81	0.35	0.729
11	2.32±1.00	1.20±0.94	3.31	0.002**
12	2.05±0.40	0.79±0.79	6.22	<0.001**
13	2.89±0.32	1.89±0.74	5.44	<0.001**
14	1.05±1.81	0.63±1.50	0.78	0.440
15	3.53±1.26	1.89±2.05	2.95	0.006**
16	1.89±0.32	1.26±0.93	2.79	0.011*
17	2.11±1.24	1.32±1.11	2.07	0.046*
18	2.11±0.57	1.21±1.08	3.19	0.04**
19	3.00±1.05	1.94±0.71	3.62	0.001*

Note: *p<0.05 **p<0.001

### Reliability of the questionnaire

Due to the questionnaires without significant difference items were analysed and deleted,

the internal consistency reliability of each sub-questionnaire and the total questionnaire were evaluated by Cronbach’ alpha coefficient. The results are shown in Table 2. The internal consistency reliability of each questionnaire is greater than 0.7, with better reliability.

**Table 2:** Cronbach ‘alpha coefficient of each questionnaire and total questionnaire

Questionnaire	Number of entries	Cronbach ‘alpha coefficient
Knowledge questionnaire	17	0.727
Attitude questionnaire	9	0.737
Behavioural questionnaire	14	0,935
Total questionnaire	40	0.861

The behavioural questionnaire includes 14 items at four dimensions: stroke knowledge acquisition and psychological nursing, health education for stroke prevention, management of risk factors, and early rehabilitation training. Using the Likert 5-point scale, it is evaluated as never=0, occasionally=1, sometimes=2, often=3, and always=4. The full score is 56 points and the lowest is 0. The higher score indicates the more positive behaviours for stroke prevention and early rehabilitation exercises (Table 3).

**Table 3:** Project analysis results of each item of neurological nurse stroke behaviour questionnaire (n=240)

Entry	High grouping	Low grouping	t	p
1	3.37±0.76	2.04±0.62	6.14	<0.001**
2	3.47±0.51	2.17±0.64	7.27	<0.001**
3	3.63±0.50	2.21±0.66	7.83	<0.001**
4	3.58±0.51	1.79±0.78	8.64	<0.001**
5	3.58±0.69	1.54±0.83	8.57	<0.001**
6	3.84±0.50	1.83±0.64	11.25	<0.001**
7	3.68±0.58	2.50±0.51	7.10	<0.001**
8	3.74±0.45	2.08±0.50	11.32	<0.001**
9	3.74±0.56	2.58±0.78	5.65	<0.001**
10	3.89±0.32	2.33±0.64	10.49	<0.001**
11	3.58±0.61	2.21±0.72	6.63	<0.001**
12	3.58±0.51	1.96±0.86	7.27	<0.001**
13	3.84±0.37	2.21±0.72	9.59	<0.001**
14	3.63±0.50	2.00±0.88	7.18	<0.001**

Note: *p<0.05 **p<0.001

## Results and discussion

The role of the neurological nurses and the demands of stroke training contents and methods were expressed in terms of frequency and percentage. The scores of strokes knowledge, attitude and behaviour were all described by mean standard deviation; independent sample t-test or one-way ANOVA^[5]^ were adopted to compare the scores of nurses’ knowledge, attitude and behaviour of nurses with different genders, working years, professional titles, education level, stroke history of relatives, employment, training and roles; bivariate Pearson correlation was used to analyse the correlation between neurological nursing staff’s stroke knowledge, attitude and behaviour scores and the correlation of numerical data; multiple linear stepwise regression analysis was made for the influencing factors on the neurologyy nurses’ stroke knowledge, attitude and behaviour, and the test standard a=0.05, with p as the two-sided probability.

### A macro model of continuous rehabilitation nursing mode for stroke patients

**Figure 1:**
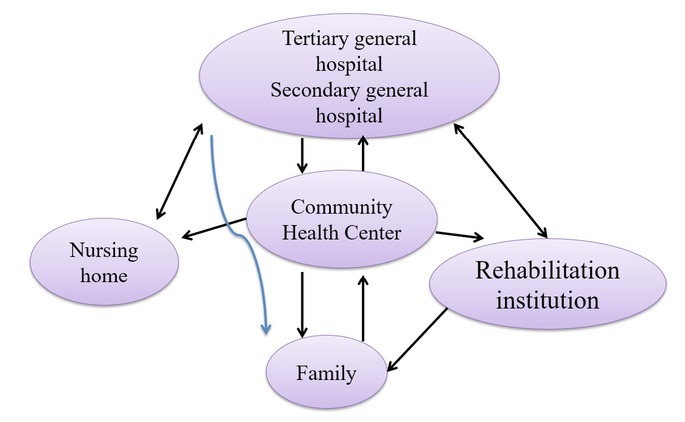
Macroscopic picture of continuous rehabilitation nursing mode for stroke patients

The responsibilities of the nurses in the continuous rehabilitation nursing service, the multi-disciplinary team collaboration mode, the continuous content of the rehabilitation nursing service and the setting of the supervision support department were revised and improved. Then, combined with the theory of continuous nursing, organizational management, self-care and the previous research results, a “continuous rehabilitation model for stroke patients” was developed (see Figure 1).

The main roles of neurology nurses are ^[6, 7]^: observational assessors of neurological patients; practitioners of rehabilitation care; coordinators of multidisciplinary teams in the therapeutic rehabilitation; managers of wards; coordinators of patient referrals.

**Figure 2:**
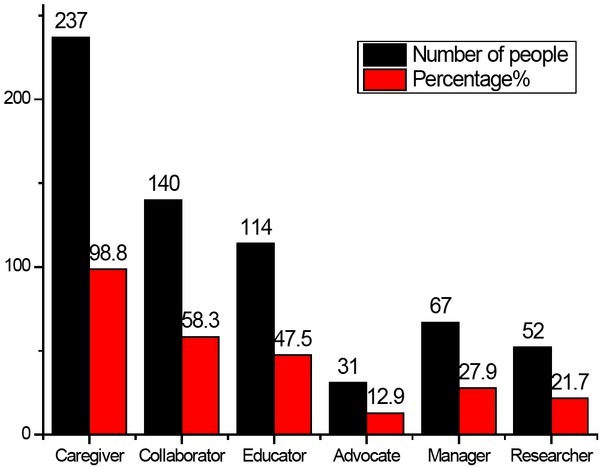
The role of neurological nurses in self-evaluation（n=240）

Nurses performed basic knowledge education for patients, including: common causes of stroke and prevention guidance, prevention and care of dysfunction and common complications, daily life guidance for stroke patients such as diet, rest, and entertainment guidance, etc.^[8, 9, 10]^, patient psychological care. They should also make records of family visits. The specific work distribution is shown in Figure 2.

From Figure 2, it can be seen that the neurological nurse mainly works as the caregiver. Only 12.9% recognized that the caregiver itself is also the patient’s spokesperson. Less than 50% could perform the role of educators in nursing work, 21.7% of nurses believe they have the role of researcher, and 27.9% of nurses believe the role of manager.

### Differences in knowledge, attitude and behavior scores of strokes among neurology nurses with similar data characteristics

### Differences in knowledge, attitude and behavior of stroke among different gender neurology nurses

Attitude of stroke is at a good level, indicating that neurological nurses’ attitude towards stroke prevention and early functional exercise is positive. The neurological nurses’ overall stroke attitude is good, showing the need of neurological nurses for stroke knowledge and training, the recognition of neurological nurses in stroke health education and early rehabilitation exercises on stroke, and the affirmation for the significance of health education and early rehabilitation exercise (Table 3).

It can be seen from Table 3 that the scores of stroke knowledge of in neurology nurses are higher than those of males, and the difference is significant (p<0.01). The female nurses’ scores in neurology department are higher than those in males, but the difference is not statistically significant (p >0.05). Neurological nurses’ stroke behaviour scores are basically the same for female and male.

In Table 4, for neurological nurses with different professional titles, the variance analysis F-value of the stroke knowledge scores is 21.225, (p<0.01), and the difference reaches significant level. Among them, the supervisor nurse has the highest scores, which is significantly different from the nurses and nurses (p<0.01). The senior nurses’ knowledge scores are higher than those of the nurses, and there is a significant difference between the two (p<0.01). Besides, there is no significant difference in the stroke knowledge score between the co-chief nurses and nurses, senor nurses, and supervisors (p>0.05).

**Table 3:** Comparison of stroke, knowledge, attitude and behaviour scores of nurses in different genders (n=240)

Gender	n	Stroke knowledge	Stroke attitude	Stroke behaviour
Male	11	30.81±5.98	29.27±6.42	40.09±11.82
Female	229	39.36±9.21	31.88±5.00	40.62±9.84
t		-3.040	-1.664	-0.173
p		0.003**	0.097	0.863

Note: *p<0.05 **p<0.001

**Table 4:** Comparison of stroke, knowledge, attitude and behaviour scores of medical staff with different professional titles (n=240)

Job title	n	Knowledge score	Attitude score	Behavioral score
Nurse	111	34.66±9.37	31.18±6.19	39.64±10.40
Head nurse	94	41.51±6.87	31.79±4.00	40.57±9.37
Supervisor	32	6.16±7.39	33.37±3.25	43.78±9.70
Deputy director nurse or above	3	42.00±14.00	34.7±2.31	42.67±6.03
F		21.225	1.904	1.499
p		<0.01**	0.130	0.216

Note: *p<0.05 **p<0.001

**Figure 3:**
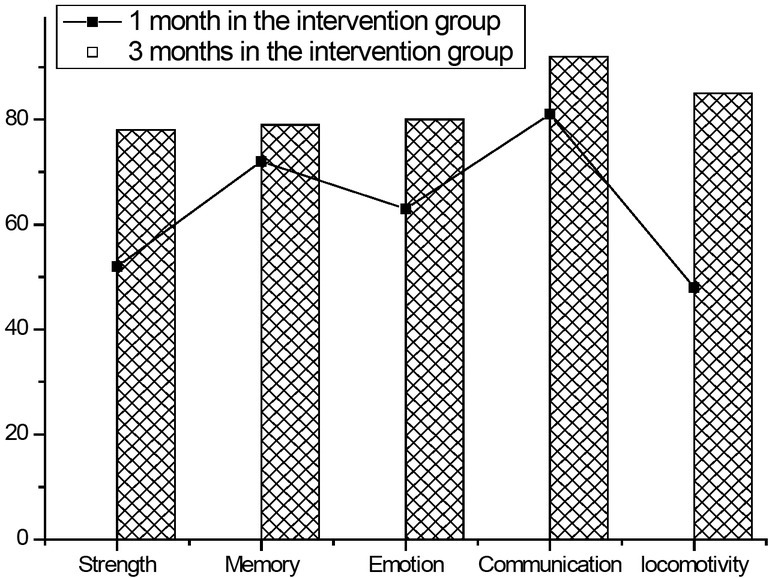
Before and after intervention, the intervention group had different dimensions of quality of life

It can be seen from Figure 3 that the effects of intervention factors and time factors on overall quality of life are statistically significant, and there is an interaction between the two factors. Over time, the effects of intervention factors on quality of life have gradually increased. The effectiveness of rehabilitation care helps nursing staffs to understand the importance of rehabilitation nursing care in the framework of nursing procedures, and to help caregivers further develop community care work.

### Differences in knowledge, attitude and practice of stroke among neurology nurses with different professional titles

It can be seen from Table 5, the knowledge scores of the neurological nurses with a history of stroke among the relatives are higher than those without the history of stroke among the relatives, and the difference is significant (p<0.01). There is no significant difference in the scores of stroke attitude among these neurological nurses with or without history of stroke among relatives (p>0.05). There is also no significant difference in stroke behaviour scores among them (p>0.05).

**Table 5:** Comparison of stroke, knowledge, attitude and behaviour scores among nursing staff with or without history of stroke in relatives (n=240)

Relatives with a history of stroke	n	Knowledge score	Attitude score	Behavioural score
Yes	83	41.48±8.11	31.86±4.29	40.96±10.05
No	157	37.64±9.7	31.71±5.48	40.40±9.86
t		3.728	0.214	0.417
p		0.001**	0.830	0.667

Note: *p<0.05 **p<0.001

The neurological nurses who participated in the stroke training has higher knowledge scores than the nurses who did not participate in the training, and there is a significant difference between the two (p<0.01). There is no significant difference in stroke scores of attitudes between neurological nurses who has or hasn’t participated in stroke training (p>0.05). There is also no significant difference in stroke behaviour scores between them (p>0.05).

## Conclusion

In summary, neurological nurses’ knowledge of stroke rehabilitation nursing increases with the years of working, the weekly special study time, and the diversity of learning styles. Therefore, the author suggests that the nursing staff should be appropriately increased to alleviate the serious shortage of nurses and improve their efficiency work. The nursing manager should arrange the work reasonably, reduce the indirect nursing work, and let the nursing staffs have more time to study professional theoretical knowledge and skills; professional training in nurses’ theoretical knowledge and skills in rehabilitation should be conducted. Training methods can be arranged according to the nurse’s job title, such as going out for further study, asking the rehabilitation doctor to give lectures and on-site guidance, conducting pre-job training for new nurses, and conducting regular continuing education to improve the comprehensive ability of nurses. Besides, rehabilitation nursing should be incorporated into the overall nursing of neurologyy, and accelerate the research on the certification system of specialist nurses in neurology department.

**Table 6:** Comparison of scores of stroke knowledge, attitudes and behaviours among medical staff who have participated in training (n=240)

Participated in stroke training	n	Knowledge score	Attitude score	Behavioural score
Yes	170	40.05±8.94	31.62±5.20	41.01±9.98
No	70	36.34±9.54	32.09±4.83	39.60±9.75
T		2.860	-0.639	0.999
p		0.005**	0.524	0.319

Note:*p<0.05 **p<0.001
